# Leucine content of dietary proteins is a determinant of postprandial skeletal muscle protein synthesis in adult rats

**DOI:** 10.1186/1743-7075-9-67

**Published:** 2012-07-20

**Authors:** Layne E Norton, Gabriel J Wilson, Donald K Layman, Christopher J Moulton, Peter J Garlick

**Affiliations:** 1 Division of Nutritional Sciences, University of Illinois at Urbana-Champaign, Urbana, IL, 61801, USA; 2 Department of Food Science and Human Nutrition, University of Illinois at Urbana-Champaign, Urbana, IL, 61801, USA; 3 Department of Animal Sciences, University of Illinois at Urbana-Champaign, Urbana, IL, 61801, USA

**Keywords:** Protein quality, Branched-chain amino acids, Whey protein, Insulin, mTOR

## Abstract

**Background:**

Leucine (Leu) regulates muscle protein synthesis (MPS) producing dose-dependent plasma Leu and MPS responses from free amino acid solutions. This study examined the role of Leu content from dietary proteins in regulation of MPS after complete meals.

**Methods:**

Experiment 1 examined 4 protein sources (wheat, soy, egg, and whey) with different Leu concentrations (6.8, 8.0, 8.8, and 10.9% (w/w), respectively) on the potential to increase plasma Leu, activate translation factors, and stimulate MPS. Male rats (~250 g) were trained for 14 day to eat 3 meals/day consisting of 16/54/30% of energy from protein, carbohydrates and fats. Rats were killed on d14 either before or 90 min after consuming a 4 g breakfast meal. Experiment 2 compared feeding wheat, whey, and wheat + Leu to determine if supplementing the Leu content of the wheat meal would yield similar anabolic responses as whey.

**Results:**

In Experiment 1, only whey and egg groups increased post-prandial plasma Leu and stimulated MPS above food-deprived controls. Likewise, greater phosphorylation of p70 S6 kinase 1 (S6K1) and 4E binding protein-1 (4E-BP1) occurred in whey and egg groups versus wheat and soy groups. Experiment 2 demonstrated that supplementing wheat with Leu to equalize the Leu content of the meal also equalized the rates of MPS.

**Conclusion:**

These findings demonstrate that Leu content is a critical factor for evaluating the quantity and quality of proteins necessary at a meal for stimulation of MPS.

## Background

Leucine (Leu) is an indispensable amino acid with a unique role in initiating protein translation. All amino acids are required as substrates for assembly of new peptides but Leu serves a second role, particularly in skeletal muscle, as a nutrient signal to initiate muscle protein synthesis (MPS). Leu functions in tandem with hormones including insulin to activate key elements of translation initiation through mTORC1 including the ribosomal protein S6 (rpS6) and the initiation factor eIF4E [1,2]. With increasing age, the contribution of anabolic hormones to initiate translation declines [3,4] increasing the importance of Leu as a post-meal anabolic signal (Rieu Nutrition 07; Glynn J. Nutr 2010; Yang Br J Nutr, 2012).

The role of the Leu signal in translation to facilitate assembly of the initiation complex has been studied with free Leu and Leu delivered with indispensable amino acid mixtures [5,6]. These studies serve to characterize the mechanism of the mTORC1 activation of MPS and established the potential for Leu to generate a post-prandial initiation signal. The role of Leu in triggering translation initiation leads to the assumption that Leu is important in defining the quantity and quality of dietary proteins at a meal necessary to stimulate MPS [7], however this hypothesis has not been well tested.

We hypothesize that the dietary impact of Leu will be greatest during conditions when MPS is down-regulated and the meal content of total protein is limited. To test this hypothesis, we used adult rats to minimize the importance of insulin-stimulated growth signals [4], a short-term food deprivation (12 h) to generate a condition of depressed translation initiation [5,8], and a small meal that was limited in both total energy and protein to optimize the importance of the Leu signal [9]. Specifically, we selected 4 food proteins (wheat gluten, soy protein isolate, egg white protein, and whey protein isolate) representing a Leu range of approximately 6.8% to 10.9% of protein (w/w) that were fed as part of a small breakfast meal providing 20% of total daily energy with protein at 16% of energy and complete profiles of macronutrients and fiber. This study demonstrates that Leu is an important factor of protein quality for translation initiation in skeletal muscle.

## Methods

### Animals and diets

Male rats (250 ± 12 g) were purchased from Harlan-Teklad (Indianapolis, IN) and maintained at 24°C with a 12-h light:dark cycle and free access to water. The animal facilities and protocol were reviewed and approved by the Institutional Animal Care and Use Committee of the University of Illinois at Urbana-Champaign.

Rats were trained for 6 day to consume 3 meals/day consisting of a 4 g meal consumed between 07:00 and 07:20 h followed by free access to food from 13:00 to 14:00 and 18:00 to 19:00 [7]. For consistency, all animals were adapted to the meal protocol using the wheat protein diet (Table 1). We have previously tested wheat and whey protein for the meal training and found that adult rats adapt to the meal protocol using either protein. After 2 day of meal-training, all rats consumed ~17 g/day of total diet equivalent to ad libitum intake. All diet treatments provided 16/54/30% of energy from protein, carbohydrates and fats, respectively.

**Table 1:** Composition of diets

**Component**	**Wheat Diet**	**Soy Diet*g/kg***	**Egg Diet**	**Whey Diet**
Vital Wheat Gluten^1^	190.2	0.0	0.0	0.0
Soy Protein Isolate^2^	0.0	185.3	0.0	0.0
Egg White Solids^3^	0.0	0.0	195.6	0.0
Whey Protein Isolate^4^	0.0	0.0	0.0	188.8
L-Lysine^5^	10.1	0.0	0.0	0.0
Cornstarch	316.7	331.7	321.4	328.2
Maltodextrin	134.1	134.1	134.1	134.1
Sucrose	101.5	101.5	101.5	101.5
Soybean Oil	140.9	140.9	140.9	140.9
Cellulose (Fiber)	53.7	53.7	53.7	53.7
Mineral Mix^6^	37.6	37.6	37.6	37.6
Vitamin Mix^6^	10.7	10.7	10.7	10.7
Choline Bitautrate	2.7	2.7	2.7	2.7
Biotin^7^	0.0	0.0	0.016	0.0

^1^Vital Wheat Gluten purchased from Honeyville Grain, Honeyville, UT. 83.4% protein, 7.6% carbohydrate, 9% other (w/w).

^2^Soy Protein Isolate provided by Archer Daniels Midland Company, Decatur, IL. 91.6% protein, 1.4% carbohydrate, 7% other.

^3^Egg White Solids purchased from Harlan-Teklad, Madison, WI. 87.8% Protein, 4.5% carbohydrate, 7.7% other.

^4^Whey Protein Isolate provided by Perham, Perham, MN. 89.9% protein, 3.8% carbohydrate, 6.3% other.

^5^Wheat Gluten supplemented with 6.3 g L-lysine/100 g protein to match Whey Protein Isolate.

^6^Mineral and vitamin mixtures [10] from Harlen-Teklad, Madison, WI.

^7^Egg White Solids supplemented with 16.0 mg biotin/kg diet.

Experiment 1 examined post-prandial changes in MPS, plasma Leu, and translation factors in rats fed meals differing in source of protein: wheat (n = 10), soy (n = 10), egg (n = 11), or whey protein (n = 11) (Table 1). All proteins exceeded minimum indispensable amino acid requirements as defined by the National Research Council (NRC) except for wheat gluten that was limiting in lysine (Table 2). Wheat gluten diet was supplemented with lysine to meet NRC requirements and to equal the lysine content of the whey protein isolate (Table 3). A baseline food-deprived control group was also adapted to meal-feeding using the wheat protein diet (n = 10). On d 6 rats were randomly assigned to groups and received their respective treatment diets for 14 day. Previous research evaluated the acute response of MPS to a single meal challenge of wheat versus whey proteins [7]. This study evaluates multiple proteins and uses an extended feeding period to determine if meal responses are maintained over a prolonged period.

**Table 2:** **Comparison of test diet amino acid compositions with NRC requirements**^**1,2**^

**Amino Acid**	**Wheat Diet**	**Soy Diet*g/kg diet***	**Egg Diet**	**Whey Diet**	**NRC Requirement**
Phenyalanine + Tyrosine	11.5	17.0	16.8	10.7	1.9
Histidine	3.1	4.2	3.9	3.4	0.8
Isoleucine	5.1	8.1	9.0	10.5	3.1
Leucine	11.5	13.6	14.9	18.5	1.8
Lysine^3^	4.7(+10.1)	10.7	11.0	15.4	1.1
Methionine + Cysteine	6.5	4.4	13.9	7.6	2.3
Threonine	4.4	6.5	7.6	10.9	1.8
Tryptophan	2.2	2.0	2.7	2.7	0.5
Valine	7.6	8.0	11.5	10.2	2.3

^1^Table 2–2 from Nutrient Requirements of Laboratory Animals Fourth Revised Edition [11].

^2^Values calculated for 300 g rat at maintenance.

^3^Vital Wheat Gluten supplemented with 10.6 g L-lysine/kg diet to match Whey Protein Isolate.

**Table 3:** Amino acid compositions of protein sources

**Amino Acid**	**Vital Wheat Gluten**^**1**^	**Soy Protein Isolate**^**2**^***g/100 g Protein***	**Egg White Solids**^**3**^	**Whey Protein Isolate**^**4**^
Alanine	3.1	4.0	6.1	4.9
Arginine	4.7	7.5	5.8	2.4
Aspartate	4.0	11.5	10.3	10.6
Cysteine	1.9	1.3	4.4	2.5
Glutamate + Glutamine	31.7	19.2	13.1	16.9
Glycine	3.8	4.1	3.5	1.8
Histidine	1.8	2.5	2.3	2.0
Isoleucine	3.0	4.8	5.3	6.2
Leucine	6.8	8.0	8.8	10.9
Lysine^5^	2.8 (+6.3)	6.3	6.5	9.1
Methionine	1.9	1.3	3.8	2.0
Phenylalanine	4.4	5.2	5.9	3.3
Proline	9.4	5.2	3.8	5.6
Serine	3.9	5.4	6.9	4.7
Threonine	2.6	3.8	4.5	6.4
Tryptophan	1.3	1.2	1.6	1.7
Tyrosine	2.4	4.8	4.0	3.0
Valine	4.5	4.7	6.8	6.0

^1^Vital Wheat Gluten purchased from Honeyville Grain, Honeyville, UT. 83.4% protein, 7.6% carbohydrate, 9% other (w/w).

^2^Soy Protein Isolate provided by Archer Daniels Midland Company, Decatur, IL. 91.6% protein, 1.4% carbohydrate, 7% other.

^3^Egg White Solids purchased from Harlan-Teklad, Madison, WI. 87.8% Protein, 4.5% carbohydrate, 7.7% other.

^4^Whey Protein provided by Perham, Perham, MN. 89.9% protein, 3.8% carbohydrate, 6.3% other.

^5^Wheat Gluten supplemented with 6.3 g L-lysine/100 g protein to match Whey Protein Isolate.

On d 15, rats were food-deprived for 12 h and then fed their normal treatment 4 g breakfast meal. The food-deprived controls received no breakfast meal. The meals provided 0, 46, 54, 60, and 74 mg of Leu for the food-deprived controls, wheat, soy, egg, and whey groups, respectively. Rats were killed 90 min after consumption of the meal and blood and tissue samples collected. Tissues were then stored at −80°C for later analyses. MPS was measured at 0 and 90-min time-points as described below.

Experiment 2 examined supplementing the wheat gluten meal with Leu to determine if matching Leu contents of the wheat and whey meals would yield similar peak rates of postprandial MPS. Based on findings in Experiment 1 and our previous research [7] demonstrating that the MPS meal response was the same after a single meal or after 14 day feeding, Experiment 2 was performed as a single meal study.

All rats were adapted to meal feeding as described in Experiment 1. After 6 day of adaptation to meal feeding, rats were assigned to treatment groups based on body weight. Animals (n = 5–6 per group) were food deprived for 12 h and then groups randomly assigned to either food deprived controls or fed one of three 4 g meals with 16% protein coming from wheat gluten, wheat gluten supplemented with Leu (wheat + Leu) (Ajinomoto, Chicago, IL) (Leu = 18.5 g/kg diet), or whey protein (Table 2). The whey protein and wheat gluten diets were supplemented with glycine (Sigma-Aldrich, St. Lois, MO) to make test meals isonitrogenous and isoenergetic with the wheat + Leu group. Post-meal responses in plasma Leu, were measured at 30, 90, and 135 min after completion of the meal to determine if supplementing free Leu altered the pattern of Leu appearance in the blood. Rats were euthanized and blood and tissues harvested as in Experiment 1. MPS was measured at 0 and 90-min time-points.

### Determination of Muscle Protein Synthesis

Protein synthesis was measured in gastrocnemius muscles using the flooding dose method [12]. A 100% enriched L-^2^ H_5_-phenylalanine solution (150 mmol/L; Cambridge Isotopes, Andover, MA) was administrated at 150 μmol/100 g body weight and injected via tail vein (1 mL/100 g body weight). After 10 min rats were killed by decapitation and hind limbs quickly removed and immersed in an ice-water mixture. Muscles were removed from cooled hind limbs, frozen in liquid N_2,_ and stored at −80°C.

Frozen muscle was powdered in liquid nitrogen and protein precipitated with cold (4°C) perchloric acid (30 g/L, 1 mL per 50 mg muscle tissue). The resulting supernatant and protein pellet were prepared for gas chromatography mass spectroscopy (GC-MS) analyzes as described previously [13,14]. Enrichment of L-^2^ H_5_-phenylalanine in the muscle hydrolysate was measured by GC-MS using a 6890 N GC and a 5973 N mass detector (Agilent Technologies Santa Clara, CA). Samples were run under electron impact ionization in splitless mode and phenylethylamine ions with mass-to-charge ratio (m/z) 106 (m + 2) and 109 (m + 5) were monitored for enrichment.

Muscle supernatants were used for determination of intracellular free phenylalanine enrichment. Free amino acids were purified by ion exchange resin solid phase extraction (SPE) using EZ:faastTM amino acid analysis sample testing kit (Phenomenex Inc. Torrance, CA, USA) and ^2^ H_5_-phenylalanine enrichment was determined using a propyl chloroformate derivative with GC-MS monitoring of ions at m/z 206 (m) and 211 (m + 5) [15].

Fractional rates of protein synthesis (MPS) were determined from the rate of incorporation of L-^2^ H_5_-phenylalanine into total mixed muscle protein as described previously [16]. The time from injection of the metabolic tracer until tissue cooling was recorded as the actual time for L-^2^ H_5_-phenylalanine incorporation. MPS, defined as the percentage of tissue protein renewed each day, were calculated according to the formula: MPS = (E_b_ x 100)/(E_a_ x t) where t is the time interval between isotope injection and snap freezing of muscle expressed in days and E_b_ and E_a_ are the enrichments of ^2^ H_5_-phenylalanine in hydrolyzed tissue protein and in muscle free amino acids, respectively.

### Plasma measurements

Plasma was obtained from trunk blood by centrifugation at 1800×*g* for 10 min at 4°C. Plasma insulin concentrations were analyzed using a commercial RIA kit for rat insulin (Linco Research, St. Charles, MO). Plasma glucose (Thermo Fisher Scientific, Middletown, VA) was determined by the glucose oxidase method. Plasma amino acid concentrations were analyzed by HPLC using a Waters 2475 Fluorescence detector [17].

### Phosphorylation of 4E-BP1, S6K1, and Akt

Muscle supernatants were subjected to protein immunoblot analyses as described previously [18,19] using rabbit polyclonal antibodies for 4E-BP1 (Bethyl Labs, Montgomery, TX), S6K1 (Bethyl Labs, Montgomery, TX), and Akt (Cell Signaling, Boston, MA).

## Statistical analysis

All data were analyzed by SPSS 15.0 (Chicago, IL) software package for Windows. For Experiment 1, a one-way ANOVA was performed with the treatment groups as the independent variables. For Experiment 2, a one-way ANOVA was performed for MPS, with treatment groups as the independent variables. Other comparisons for Experiment 2 utilized a 3 × 4 (*i.e.* experimental groups: wheat, wheat + Leu, and whey x time: 0, 30, 90, and 135 min) repeated measures ANOVA to determine within and between group differences. When a significant overall effect was detected, differences among individual means were assessed using Fisher’s LSD post hoc test. Data sets were tested for normal distribution and variance homogeneity using Levene’s test. When variances were not homogeneous, means were compared using a Games-Howell test. Significance was set at P < 0.05 for all statistical tests. All values are presented as means ± SEM.

## Results

### Experiment 1

This experiment compared isonitrogenous, isoenergetic meals containing wheat gluten, soy protein isolate, egg white protein, or whey protein isolate as the protein source on the potential to initiate translation and protein synthesis in skeletal muscle. After consumption of a 4 g meal providing 20% of daily energy and containing 16% of energy as protein, plasma Leu increased in egg or whey groups but not in wheat or soy groups. Similar post-prandial patterns occurred for each of the BCAA with whey producing the highest concentrations at 90 min after the meal (*i.e.* whey > egg > soy > wheat) (Table 4).

**Table 4:** **Experiment1: Selected plasma essential amino acid**^**1**^**and glucose**^**2**^**concentrations 90 min after feeding meals containing wheat, soy, egg, or whey proteins**^**3-4**^

	**Baseline**^**5**^	**Wheat**	**Soy**	**Egg**	**Whey**
Leucine	84 ± 4.6^c^	78 ± 4.3^c^	84 ± 5.6^c^	146 ± 8.4^b^	192 ± 11.4^a^
Isoleucine	56 ± 3.4^d^	50 ± 2.9^d^	74 ± 4.0^c^	121 ± 6.3^b^	144 ± 8.2^a^
Valine	117 ± 8.2^cd^	95 ± 5.2^d^	143 ± 8.1^c^	295 ± 14.2^b^	248 ± 13.7^a^
∑ BCAA	257 ± 16.0^bc^	223 ± 12.1^c^	301 ± 17.4^b^	562 ± 28.5^a^	584 ± 33.0^a^
Lysine	510 ± 29.8^ab^	527 ± 23.8^ab^	419 ± 37.2^b^	495 ± 35.6^ab^	549 ± 29.3^a^
Methionine	51 ± 2.9^bc^	46 ± 2.8^bc^	38 ± 4.1^c^	86 ± 6.8^a^	52 ± 5.2^b^
Threonine	252 ± 13.7^c^	269 ± 30.4^bc^	349 ± 40.6^bc^	357 ± 28.3^ab^	538 ± 51.2^a^
Glucose	7.8 ± 0.6^b^	9.9 ± 0.5^a^	8.5 ± 0.7^ab^	9.7 ± 0.8^a^	9.4 ± 0.4^a^

^1^Plasma amino acids expressed as *μmol/L.*

^2^Plasma glucose expressed as *mmol/L.*

^3^Values expressed as means ± SEM, n = 8-10. Means without a common letter differ (P < 0.05).

^4^12 h food-deprived controls.

Other plasma amino acids varied among the groups largely in proportion to the amino acid content of the protein source (Table 4). Lysine was different between soy and whey treatments, with the whey group having the greatest concentration of post-prandial lysine and the soy group having the lowest lysine levels. The plasma concentration for the wheat group reflects the lysine supplementation of the diet. Post-prandial plasma methionine concentrations were increased with the egg treatment while other groups were not different from food deprived controls (Table 3). Plasma threonine was increased after the meal for egg or whey groups with highest concentrations observed in rats consuming the whey protein.

**Figure 1:**
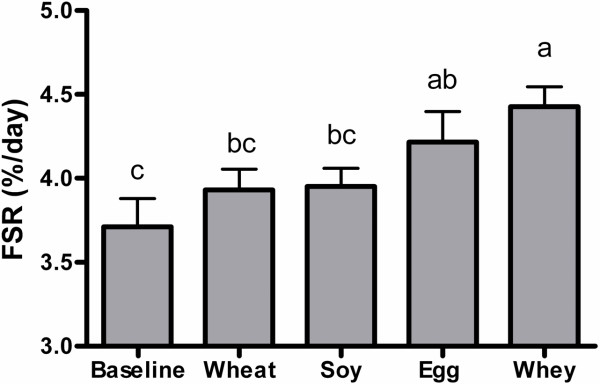
** Experiment 1 Muscle Protein Synthesis.** Rates of protein synthesis in gastrocnemius muscle of rats after consuming complete meals containing wheat, soy, egg, or whey proteins. Data are means ± SEM; n = 9–10. Labeled means without a common letter differ, P < 0.05.

**Figure 2:**
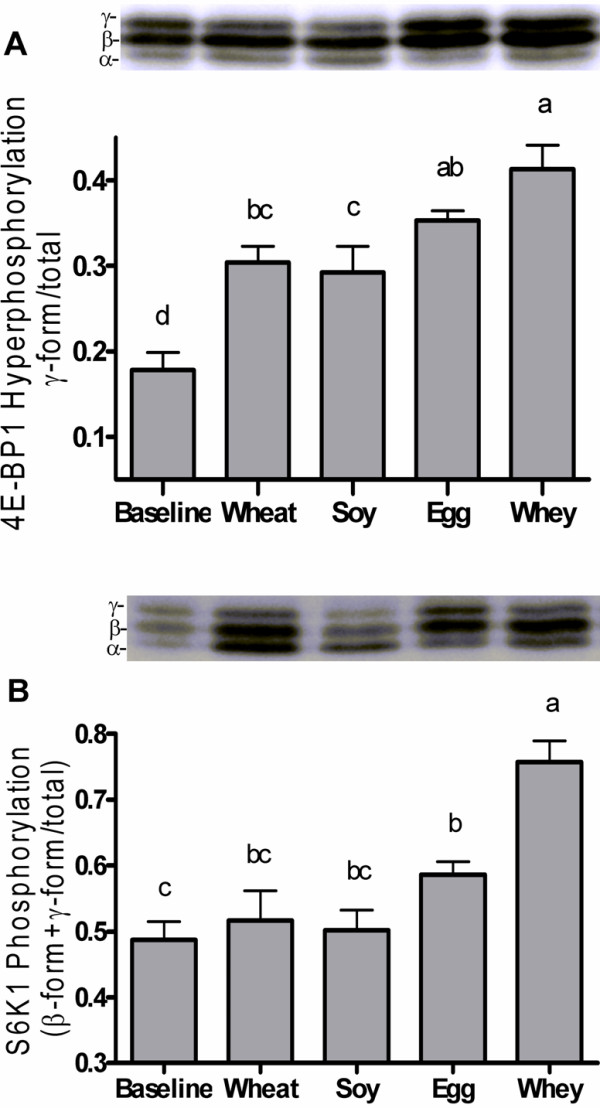
** Experiment 1 Translation Factor Activation.** Phosphorylation states of 4E-BP1 (**A**) and p70S6K1 (**B**) in gastrocnemius muscle of rats after consuming meals containing wheat, soy, egg, or whey proteins. Data are means ± SEM; n = 9–10. Labeled means without a common letter differ, P < 0.05.

After the meal, MPS increased in the egg or whey groups with the highest value observed for the whey group (Figure 1). The MPS response was consistent with phosphorylation of the mTORC1 signaling targets 4E-BP1 (Figure 2A) and S6K1 (Figure 2B). Phosphorylation of S6K1 increased after egg or whey meals but not wheat or soy with peak values obtained in the whey protein group. 4E-BP1 phosphorylation increased in all groups with whey greater than wheat or soy and egg greater than soy but not different from wheat or whey.

**Figure 3:**
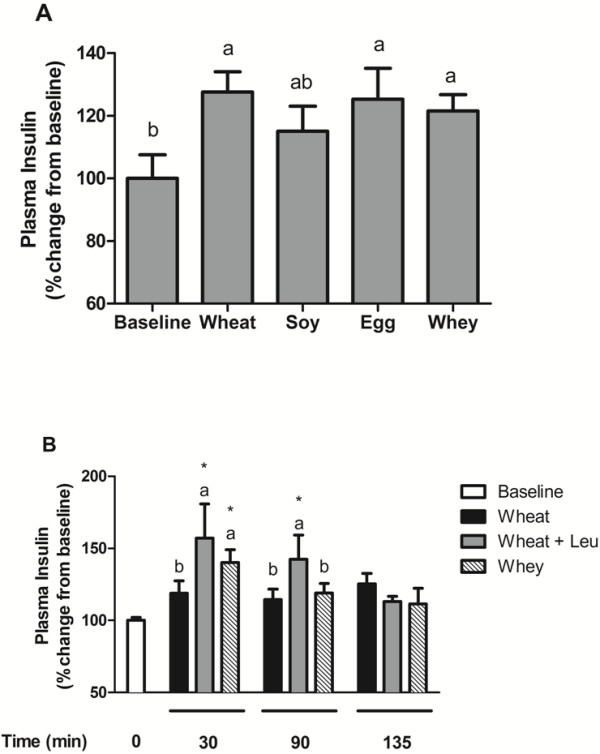
** Experiment 1 and 2 Plasma Insulin.** Changes in plasma insulin from baseline in rats after **A**) consuming meals containing wheat, soy, egg, or whey proteins or **B**) rats consuming isonitrogenous meals containing wheat, wheat supplemented with leucine (Wheat + Leu), or whey protein. Data expressed expressed as percent change from baseline and are means ± SEM; n = 5–10.

Plasma insulin increased above baseline at 90 min after the meal in all groups except soy, which was not different from food-deprived controls (Figure 3). Post-prandial insulin was not significantly different between any of the treatment groups. Similarly, plasma glucose at 90 min was increased above baseline concentrations in all groups except for soy. The glucose concentration for the soy group was not different from baseline or the wheat, egg, or whey groups (Table 4).

**Figure 4:**
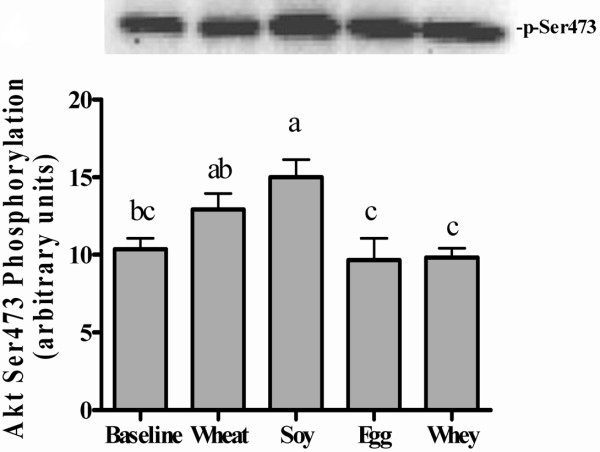
** Experiment 1 Akt Phosphorylation.** Phosphorylation states of Akt in gastrocnemius muscle of rats after consuming meals containing wheat, soy, egg, or whey proteins. Data are means ± SEM; n = 9–10. Labeled means without a common letter differ, P < 0.05.

Akt activation (ie. phosphorylation at Ser473) was increased at 90 min in the soy group compared with food deprived controls rats or the egg or whey groups (Figure 4). The wheat group had intermediate levels of Akt phosphorylation that was not different from baseline or soy groups but greater than egg or whey groups.

### Experiment 2

**Figure 5:**
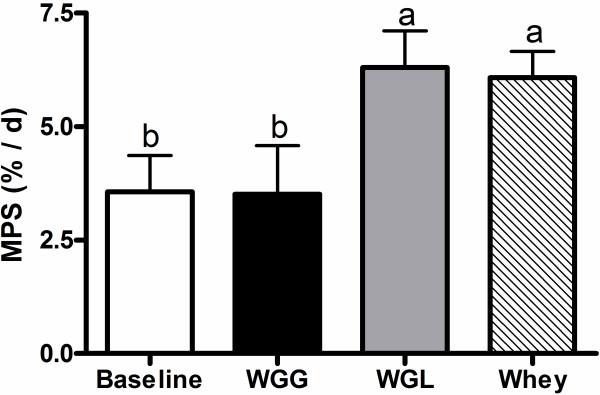
** Experiment 2 Muscle Protein Synthesis.** Rates of protein synthesis in gastrocnemius muscle of rats consuming isonitrogenous meals containing wheat,wheat supplemented with leucine (Wheat + Leu), or whey protein. Data are means ± SEM; n = 5–6. Labeled means without a common letter differ, P < 0.05.

Experiment 2 compared meals containing wheat gluten, wheat + Leu, or whey proteins on the potential to increase plasma Leu and MPS over a 135-min time-course. At all time-points, wheat + Leu and whey groups had greater increases in plasma Leu than wheat or food-deprived controls; but wheat + Leu and whey were not different from each other (Table 5). Consistent with the plasma Leu changes, MPS increased in whey and wheat + Leu groups but not in the wheat group and were not different from each other (Figure 5).

**Table 5:** **Experiment 2: Post-prandial changes for plasma amino acids**^**1-3**^

**Baseline**		**Whey**			**Wheat**			**Wheat + Leu**		
**Time (min)**		**30**	**90**	**135**	**30**	**90**	**135**	**30**	**90**	**135**
Leu	86 ± 4	226 ± 17^a*^	164 ± 26^a*^	173 ± 22^a*^	151 ± 8^b*^	86 ± 6^b^	99 ± 5^b^	211 ± 8^a*^	137 ± 8^a*^	148 ± 3^a*^
Ile	69 ± 2	166 ± 11^a*^	104 ± 4^a*^	134 ± 16^a*^	110 ± 6^b*^	66 ± 3^b^	86 ± 6^b^	98 ± 6^b*^	60 ± 4^b*^	67 ± 1^c^
Val	117 ± 5	234 ± 17^a*^	161 ± 5^a*^	186 ± 19^a*^	154 ± 8^b*^	91 ± 3^b^	104 ± 7^b^	131 ± 18^b^	77 ± 6^b*^	77 ± 2^c*^
Lys	608 ± 24	1083 ± 78^*^	593 ± 34	688 ± 62	930 ± 64^*^	553 ± 28	698 ± 14	933 ± 67	597 ± 55	726 ± 48
Met	49 ± 2	102 ± 6^a*^	62 ± 2^a*^	80 ± 5^a*^	72 ± 3^b*^	42 ± 1^b^	52 ± 3^b^	71 ± 3^b*^	44 ± 4^b^	46 ± 2^b^
Thr	309 ± 9	594 ± 73^*^	567 ± 18^a*^	554 ± 38^a^	383 ± 21	330 ± 18^b^	314 ± 22^b^	387 ± 12	382 ± 20^ab^	308 ± 13^b^

^1^Plasma amino acids expressed as *μmol/L*.

^2^Data are means ± SEM; n = 5−6. Means without a common letter differ between treatments within.

time-points, *P* < 0.05.* Indicates different from fasted (*P* < 0.05).

^3^12 h food-deprived controls.

Plasma amino acid concentrations for the wheat and whey groups were consistent with Experiment 1. The wheat + Leu group was similar to wheat except Ile and Val concentrations tended to be lower in the Leu supplemented group. The time course patterns of change in plasma amino acids were similar for all groups. The amplitude of the post-prandial changes varied largely according to the amino acid content of the diet with the highest concentrations observed at 30 min declining at 90 and 135 min.

Post-prandial insulin concentrations at 30 min were greater in whey and wheat + Leu groups compared with the wheat group (Table 5). Akt signaling tended to increase from fasted at 30 min in both whey and wheat + leu groups but did not reach statistical significance at any time point (data not shown).

## Discussion

Leu is recognized as a signaling factor for translation initiation and MPS [5,18,20]. However, the majority of this research has been done with large acute doses of free Leu, and the significance of this signal in physiological meals with different dietary proteins remains largely unknown. Accordingly, the present study examined responses of plasma amino acids, translation initiation factors, and MPS in adult rats fed complete meals with protein sources selected for differences in Leu content. This study demonstrated that in small meals with limited protein intake, MPS was stimulated in proportion to activation of translation factors and further establishes a threshold requirement for a minimum Leu content within a meal.

The relationship between post-prandial plasma Leu concentrations and stimulation of MPS has been previously observed with both animal [7,21] and human [22,23] studies. However, other studies reported using supplemental Leu producing substantial increases in plasma Leu that had no incremental effect on MPS [24-26]. While these studies appear contradictory, taken together, these studies are consistent with the hypothesis that Leu acts as a threshold trigger for activation of translation initiation. As plasma Leu increases after a meal from reduced fasted concentrations to post-prandial peak values, the change in concentration is recognized by the mTORC1 signal complex and triggers initiation. Once the threshold for activation has been achieved and the active ribosome complex assembled, additional increments in plasma Leu have minimal effects [5,7].

Norton *et al.*[7] conducted a dose response study examining wheat and whey proteins fed at 10%, 20% and 30% of dietary energy. At isonitrogenous intakes, whey protein produced higher levels of plasma Leu, mTOR signaling, and MPS than wheat. However, MPS was not different comparing meals with 30% wheat vs. 20% whey consistent with the net Leu available from wheat (6.8% Leu) and whey (10.9% Leu). Similar support for Leu as a signal to initiate an anabolic meal response has been observed for young men consuming whey, soy and casein after exercise [23] and in older men or rats consuming whey versus casein [21,22].

To further test the physiological significance of the threshold hypothesis, Experiment 1 utilized a meal-feeding protocol with adult rats. Based on previous research with rats of the same age and size [7] we estimated that the Leu required to achieve the initiation signal threshold at a meal would be ~50 to 60 mg of Leu. We selected 4 proteins that provided a range of Leu contents and designed a meal to provide 20% of daily energy and 16% of energy as protein. This combination of meal size and protein content allowed the 4 proteins to bracket the proposed Leu threshold (ie. wheat 46 mg, soy 54 mg, egg 60 mg, and whey 74 mg of Leu). The whey and egg proteins provided sufficient Leu to increase plasma Leu concentrations, increase phosphorylation of translation factors S6K1 and 4E-BP1, and stimulate MPS. These findings support a concept for a minimum meal threshold for Leu to stimulate post-prandial MPS.

While dietary Leu appears to be a critical factor to account for the post-meal MPS response, this does not exclude potential contributions from other amino acids. There is evidence that orally administered isoleucine (Ile) also activates mTORC1 signaling, although not as potent as Leu [18]. In complete proteins, Ile is typically present in proportion to the Leu content, allowing the possibility that Ile may in part contribute to the mTORC1 signaling and MPS. However, in the current study, rats fed soy significantly increased post-prandial plasma Ile concentration with no corresponding increase in mTORC1 signaling or MPS. This is consistent with previous research that only Leu among the EAA was able to increase MPS when infused in rats [27]. In the present study with complete meals, only the post-prandial changes in plasma Leu predicted changes in mTORC1 signaling and stimulation of MPS.

Beyond Leu and Ile contents, dietary proteins differ in other characteristics including distribution of other indispensable amino acids, differences in gastric emptying or digestibility, and insulinogenic properties. Experiment 2 was designed to isolate the importance of Leu within a meal and to test the significance of Leu content versus other intrinsic properties of protein sources. Experiment 2 demonstrated that supplementing the wheat protein meal with Leu to equalize the Leu content of the meals (wheat + Leu = whey) produced similar post-prandial patterns for plasma Leu and MPS response. Further, for each of the treatment groups, peak plasma Leu concentration occurred 30 min after the meal, and there was a strong correlation with Leu content of the meal and peak plasma Leu concentration (*r* = 0.919; *P* < 0.05). These findings are consistent with the post-exercise data of Churchward-Venne et al. [28] demonstrating that a suboptimal dose of whey protein (6.25 g) supplemented with Leu could produce the same MPS rate as a high dose of whey protein (25 g). These studies confirm that between different protein sources Leu content is an important predictor of post-meal MPS responses.

Also supporting the Leu threshold hypothesis, there are numerous examples of saturation of the Leu signal [5,7,19]. Using oral doses of free Leu ranging from 0.068 g/kg body weight up to 1.35 g/kg, Crozier et al. [5] demonstrated that the maximum rate of MPS was achieved in a 200 g rat at ~67 mg of Leu and that this represented maximum activation of translation initiation signals. Increasing dosage of Leu to double and triple this amount proportionally increased plasma Leu concentration but had no additional effects on initiation factors or MPS. Likewise, Debras et al. [24] observed no difference in MPS after a casein-based meal containing 81 mg of Leu versus casein plus Leu supplement with 237 mg of Leu; Norton et al. [7] found that a meal with 30% of energy from wheat gluten was similar to a 20% whey protein meal in producing maximum MPS and translation initiation responses; and Anthony et al. [19] reported that rats fed either a 20% soy or 20% whey protein meal after exercise both increased plasma Leu to approximately double baseline concentrations and both diets fully stimulated mTORC1 signaling and MPS. In total, these studies demonstrate that Leu is an essential threshold signal for translation initiation but additional amounts above the threshold do not produce additive effects on activation.

The current studies demonstrate that insulin does not differentiate regulation of post-prandial MPS in adult rats consuming that same amounts of total carbohydrates and protein. Prior research suggests that insulin plays a permissive role in Leu-dependent stimulation of protein synthesis and translation initiation [8]. Consistent with this view, all protein groups in the current studies exhibited post-prandial increases in insulin and/or the down-stream signal element Akt-phosphorylation allowing for a permissive anabolic response to the meal, however the precise insulin concentration or the activation state of Akt had no apparent relationship to absolute rates of MPS. In the current studies, the post-prandial insulin response was relatively small presumably due to meal-feeding protocol and restricted carbohydrate load of the test meals. In Experiment 2, the insulin response was greatest 30 min post-meal in the whey and wheat + Leu groups relative to baseline control and wheat groups consistent with increases in MPS. While the test meals contained equal quantity and quality of carbohydrates and equal amounts of protein, the heightened insulin response in whey protein and wheat + Leu fed rats may contribute to greater rates of mTOR signaling and MPS. The specific contribution of insulin to MPS regulation in adults remains to be fully elucidated.

## Conclusions

This research demonstrates that when protein is limited within the context of a small meal stimulation of MPS is dependent on the availability of sufficient Leu to initiate translation. Leu serves as a signal to facilitate assembly of the translation initiation complex [2]. Leu serves as a trigger to allow MPS to transition from a depressed state characterized with inhibition of translation factors 4E-BP1 and S6K1 after an overnight fast, to an active period of MPS. Hence, while the total daily protein intake may satisfy dietary guidelines, individual small meals with limited protein quantity and proteins with low Leu content may be inadequate to initiate the assembly process and stimulate MPS. This research supports the hypothesis that the Leu content of dietary proteins is important for muscle protein synthesis and highlights the need for long-term studies examining the impact of Leu density on muscle health and body composition.

## Abbreviations

4E-BP1: Eukaryotic initiation factor 4E binding protein-1; AMPK: AMP Kinase; EAA: Essential amino acids; eIF: Eukaryotic initiation factor; FSR: Factional rate of protein synthesis; Leu: Leucine; Ile: Isoleucine; MPS: Skeletal muscle protein synthesis; mTOR: Mammalian target of rapamycin; S6K: Ribosomal protein p70 S6 Kinase.

## Competing interests

L.E. Norton scientific consultant to Scivation Inc., G.J. Wilson, C.J. Moulton, and P.J. Garlick, no conflicts of interest. D.K. Layman participates in NDC and National Cattleman’s Beef Association speaker bureaus and serves on ENC Scientific Advisory Panel.

## Authors’ contributions

LEN, GJW, CJM, and DKL designed research; LEN, GJW, CJM, and PJG conducted research; LEN analyzed data; LEN, GJW, CJM, and DKL wrote the paper. LEN had primary responsibility for final content. All authors read and approved the final manuscript.
